# Astrocytes derived from glial-restricted precursors promote spinal cord repair

**DOI:** 10.1186/jbiol35

**Published:** 2006-04-27

**Authors:** Jeannette E Davies, Carol Huang, Christoph Proschel, Mark Noble, Margot Mayer-Proschel, Stephen JA Davies

**Affiliations:** 1 Department of Neurosurgery, Baylor College of Medicine, 1709 Dryden Street, Suite 750, Houston, Texas 77030, USA; 2 Department of Neuroscience, 1 Baylor Plaza, Houston, Texas 77030, USA; 3 Department of Biomedical Genetics, University of Rochester Medical Center, 601 Elmwood Avenue, Rochester, New York 14642, USA

## Abstract

**Background:**

Transplantation of embryonic stem or neural progenitor cells is an attractive strategy for repair of the injured central nervous system. Transplantation of these cells alone to acute spinal cord injuries has not, however, resulted in robust axon regeneration beyond the sites of injury. This may be due to progenitors differentiating to cell types that support axon growth poorly and/or their inability to modify the inhibitory environment of adult central nervous system (CNS) injuries. We reasoned therefore that pre-differentiation of embryonic neural precursors to astrocytes, which are thought to support axon growth in the injured immature CNS, would be more beneficial for CNS repair.

**Results:**

Transplantation of astrocytes derived from embryonic glial-restricted precursors (GRPs) promoted robust axon growth and restoration of locomotor function after acute transection injuries of the adult rat spinal cord. Transplantation of GRP-derived astrocytes (GDAs) into dorsal column injuries promoted growth of over 60% of ascending dorsal column axons into the centers of the lesions, with 66% of these axons extending beyond the injury sites. Grid-walk analysis of GDA-transplanted rats with rubrospinal tract injuries revealed significant improvements in locomotor function. GDA transplantation also induced a striking realignment of injured tissue, suppressed initial scarring and rescued axotomized CNS neurons with cut axons from atrophy. In sharp contrast, undifferentiated GRPs failed to suppress scar formation or support axon growth and locomotor recovery.

**Conclusion:**

Pre-differentiation of glial precursors into GDAs before transplantation into spinal cord injuries leads to significantly improved outcomes over precursor cell transplantation, providing both a novel strategy and a highly effective new cell type for repairing CNS injuries.

## Background

Traumatic injury to the adult central nervous system (CNS) is associated with multiple different types of damage, all of which pose substantial challenges to attempts to carry out tissue repair. Promoting regenerative growth of severed motor and sensory axons requires the provision of appropriate substrates and/or the overriding of a variety of inhibitors that prevent axon regeneration. The expression of molecular inhibitors of axon growth has been extensively characterized in fibrotic, glial scar tissue [1-4] and in CNS myelin [5-7]. In particular, adult astrocytes at sites of injury have been shown to express proteoglycans that inhibit axon growth [4,8,9] and have a major role in the formation of misaligned scar tissue [10], which lacks the linear organization of adult CNS white matter thought to be required for rapid, long-distance axon growth [11-14].

A wide range of approaches have now been applied following CNS injury to promote regenerative growth of both sensory and motor axons, with a particular focus on the transplantation of a variety of cell types, often in combination with other therapies. Cell-based transplantation strategies for promoting axon growth across spinal cord injuries [15] have included the use of neural stem cells, neonatal brain astrocytes, fibroblasts, bone-marrow derived cells and peripheral nervous system glia such as Schwann cells and olfactory ensheathing cells. Although transplants of some cell types have provided more benefit than others, the general lack of significant axon regeneration beyond sites of injury has led to the combination of cellular transplant strategies with delivery of neurotrophic factors, treatments designed to override or degrade the scar, and/or with the use of biomaterials to offer both potential substrates and organized tissue structures [16,17]. Such combinations have resulted in varying degrees of successful axon regeneration.

We have been interested in the possibility that repair of adult CNS injuries might be particularly enhanced with the introduction of cells from the immature CNS, a tissue that has a far greater regenerative capacity than the adult CNS (reviewed in [18]). One possible approach is to transplant embryonic stem cells or neural progenitor cells. Although these cells have been shown to promote limited behavioral recovery via remyelination of host axons [19-22], their transplantation directly into or adjacent to traumatic spinal cord injuries has not resulted in the regeneration of significant numbers of endogenous axons across the site of injury [21,23-25]. This may be due to the failure of the majority of these cells to differentiate [26] or because the inflammatory environment of adult CNS injuries directs undifferentiated neural stem cells or glial progenitors to a 'scar astrocyte'-like phenotype [27] that is poorly supportive of axon growth [8,28].

An alternative to allowing the lesion environment to regulate differentiation of stem or progenitor cells is to transplant a cell type from the immature CNS that is known to be supportive of axon growth. In this regard, embryonic astrocytes have long been thought of as an attractive cell type for repair of the adult CNS [29]. Establishing astrocytic cultures directly from the embryonic CNS, however, generates cell populations containing mixed astrocytic phenotypes contaminated with glial progenitors and microglia, and such populations have yielded relatively modest success in promoting axon regeneration after transplantation to adult CNS injuries [30,31]. Isolating embryonic astrocytes directly from the embryonic CNS is also very challenging, owing to the relatively low abundance of these cells *in vivo*. The generation of postnatal astrocytic cultures is normally associated with prolonged growth in conditions *in vitro *that allow aging of these cells to a less supportive phenotype [32], which has also resulted in minimal axon growth after their transplantation to adult spinal cord injuries [33].

To address the above problems, we have explored the alternative approach of pre-differentiating embryonic glial precursors to specific astrocytes *in vitro*, a technique that permits the rapid generation of sufficiently large, homogeneous populations of embryonic astrocytes of a desired phenotype for transplantation to adult CNS injuries. In applying this approach, we have generated pure populations of astrocytes directly from glial-restricted precursor (GRP) cells [34-36], the earliest arising progenitor cell population restricted to the generation of glia. Astrocytes were generated by exposing GRP cells to bone morphogenetic protein-4 (BMP-4), which induces astrocyte generation from embryonic neural precursor cells and GRP cells both *in vitro *and *in vivo *[34,37] and is thought to have important roles in regulating glial differentiation *in vivo *[38]. GRP-derived astrocytes (GDAs) generated by BMP exposure fall within the population of cells defined by their antigenic phenotype as type-1 astrocytes. Studies *in vitro *of type-1 astrocytes purified from the postnatal CNS have shown that they promote extensive neurite growth from a variety of neurons [39,40], express high levels of molecules that support axon growth, such as laminin and fibronectin [41] and nerve growth factor (NGF) or neurotrophin-3 (NT-3) [42] and also show minimal immunoreactivity to chondroitin sulfate proteoglycans (CSPG) [41]. Moreover, the directed generation of astrocytes from embryonic GRP cells may provide cells that show the beneficial axon-growth-promoting properties that characterize the early CNS.

Our study shows that transplantation of GDAs into acute spinal cord injuries promotes levels of tissue reorganization, axon regeneration and locomotor recovery that previously have been achieved only by combining cell transplantation with multiple therapeutic approaches. We also show, in identical lesions, that transplanted GRP cells are not supportive of axon growth or functional recovery, thus demonstrating the critical importance of pre-differentiating progenitor cells before transplantation to the injured adult CNS.

## Results

### Regeneration of endogenous dorsal column axons

**Figure 1:**
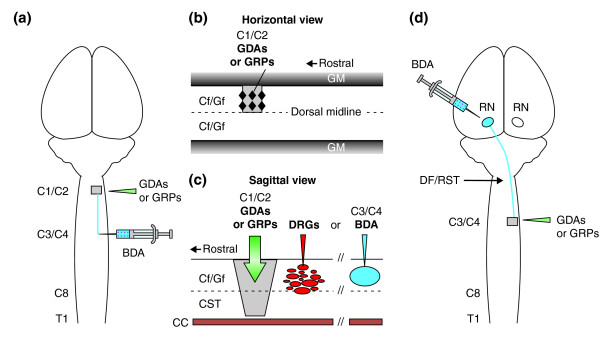
The models of spinal cord injury in adult rats used in this study. Schematic illustrations of **(a-c) **white matter of the dorsal column and **(d) **the dorsolateral funiculus white matter pathways of the spinal cord. (a,d) Dorsal views of the rat brain and spinal cord. (b) horizontal and (c) sagittal views of the dorsal column white matter pathways at the C1/C2 cervical vertebrae of the spinal cord. (a) Dorsal column white matter on the right side was transected (shaded area) at the C1/C2 spinal level, and the ability of either BDA-labeled endogenous axons or axons from microtransplanted GFP-expressing adult sensory neurons (DRGs) to cross injuries bridged with GDAs or GRPs was assayed. (b) Injections of GDA or GRP cells (black diamonds) suspended in medium were made directly into the centers of the injury sites as well as their rostral and caudal margins in the cervical spinal cord. (c) A discreet population of endogenous ascending axons within the cuneate and gracile white matter pathways of dorsal columns was labeled by BDA injection at the C3/C4 spinal level (5 mm caudal to the lesion site, shaded). Alternatively, microtransplants of GFP^+ ^DRGs were injected 500 μm caudal to the injury site. (d) The right-side dorsolateral funiculus white matter containing descending axons of the rubrospinal tract was transected at the C3/C4 spinal level and GDAs or GRPs were transplanted as described for dorsal column injuries. To trace axotomized rubrospinal tract axons, BDA was injected into the left-side red nucleus (RN) 8 days before the end of each experiment. CC, central canal; Cf, cuneate fasciculus; CST, corticospinal tract; DF, dorsolateral funiculus; Gf, gracile fasciculus; GM, gray matter; RST, rubrospinal tract; T1, level of the first thoracic vertebra.

**Figure 2:**
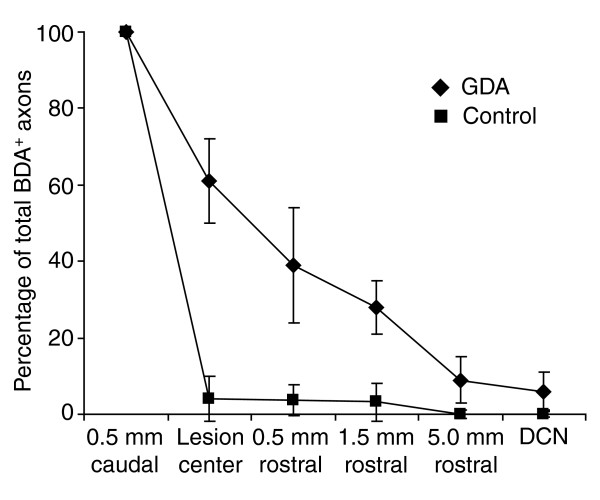
Quantification of numbers of regenerating BDA^+ ^axons in GDA-transplanted versus control dorsal column white matter at 8 days after injury and transplantation. BDA-labeled axons were counted in every third sagittally oriented section within the lesion center and at points 0.5 mm, 1.5 mm, and 5 mm rostral to the injury site, up to and including the dorsal column nuclei (DCN). Note that 61% of BDA^+ ^axons had reached the centers of GDA-transplanted lesions and 39% to 0.5 mm beyond injury sites, compared with just 4% (lesion center) and 3.8% (0.5 mm rostral) present in controls. The steady decline in numbers of BDA^+ ^axons within rostral white matter indicates a staggered front of maximum axon growth beyond sites of injury in GDA-transplanted groups at this time point. Note the total absence of axons at 5.0 mm rostral and in dorsal column nuclei in controls. Counts of BDA^+ ^axons labeled in all adjacent sagittally oriented sections in representative GDA-treated and control lesioned cords revealed totals of 372 and 330 axons, respectively, at 0.5 mm caudal to the injury site. Increases in numbers of BDA^+ ^axons in GDA-treated animals compared with controls were statistically significant (*p *< 0.01) in all rostral spinal cord regions. Error bars indicate ± 1 standard deviation.

**Figure 3:**
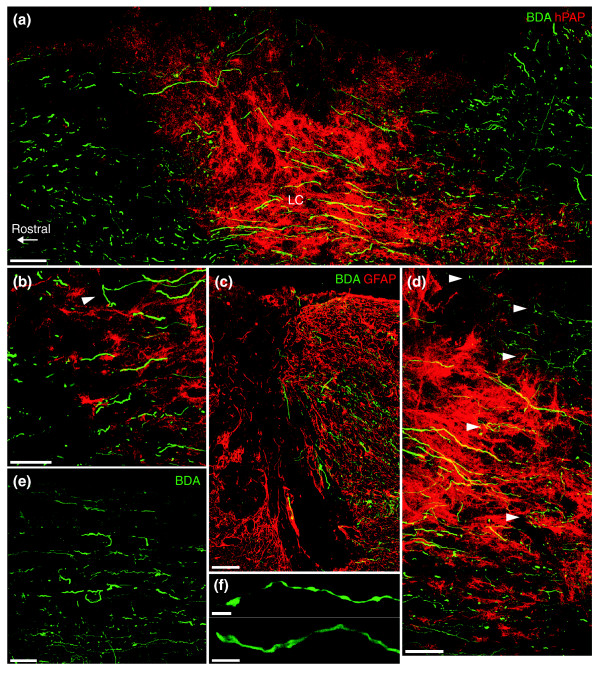
Endogenous sensory axon regeneration across GDA-transplanted dorsal column injuries at 8 days after lesion and transplantation. **(a) **A montaged, low-magnification confocal image scanned from a single 25-μm thick sagittal section, showing BDA-labeled ascending dorsal column axons (green) that have entered, grown within and exited a hPAP^+ ^(red) GDA-transplanted dorsal column lesion. LC, lesion center. **(b) **A high-magnification image of a rostral graft/host interface showing BDA^+ ^axons exiting the GDA graft and entering host white matter. A few axons were observed to have turned away from the interface and grown back towards the lesion center (arrowhead). **(c) **In control lesions, the vast majority of BDA^+ ^axons have formed dystrophic endings and failed to leave the caudal margins of the lesion, marked by hypertrophic GFAP^+ ^astrocytes (red). **(d) **A high-magnification image showing numerous BDA^+ ^axons that have successfully crossed the host/graft interface at the caudal lesion margin. A few cut axons (arrowheads) have, however, failed to leave the caudal lesion interface and can be seen to have turned and/or formed dystrophic endings, particularly in regions containing few hPAP^+ ^GDAs (red). **(e) **BDA^+ ^axons located near the pial surface and ventral regions of cuneate white matter at 1.5 mm rostral to a GDA-bridged lesion site. **(f) **BDA^+ ^axon growth cones in white matter 1.5 mm rostral to the lesion site often display streamlined growth cones indicative of rapid growth. Scale bars: (a,c) 100 μm; (b-e) 50 μm; (f) 5 μm (top) and 10 μm (bottom).

Transplantation of GDAs into stab-wound lesions of the dorsal column white matter pathways of adult rat spinal cord (Figure 1a–c) resulted in the growth of the majority of the cut ascending dorsal column axons into the lesion center (Figures 2 and 3a), with 66% of these axons extending further beyond the lesion site into adjacent white matter (Figures 2 and 3a,b,e,f). In order to minimize labeling of spared axons, a discrete population of ascending axons aligned with the lesion site was traced *en passage *with a single biotinylated dextran amine (BDA) injection caudal to GDA-transplanted or control stab injuries of the right-hand dorsal column cuneate and gracile white matter pathways (Figure 1c; see the Glossary box for an explanation of terms). Previous studies have shown that about 30–40% of ascending dorsal column axons projecting to the dorsal column nuclei arise from postsynaptic dorsal column neurons in spinal laminar 4 and that 25% of ascending dorsal column axons are also propriospinal in origin [43,44]. Indeed, it is thought that only 15% of primary afferents of dorsal root ganglion (DRG) neurons entering the spinal cord at lumbar levels reach the cervical spinal cord and that most leave dorsal column white matter within two to three segments of entering [45]. Therefore our *en passage *labeling of dorsal column axons at the cervical level included significant proportions of axons from both DRG neurons and CNS spinal neurons.

Sample counts from every third parasagittal section at 8 days after injury revealed similar numbers of BDA-labeled axons 0.5 mm caudal to the injury site in both control and experimental spinal cords, with averages of 107 ± 47 versus 101 ± 45 axons sampled per animal, respectively (see also Figure 2). In GDA-transplanted cords, on average 61% (standard deviation (s.d.) ± 11) of caudal labeled axons extended into the lesion center, 39% (s.d. ± 15) of caudal axons extended 0.5 mm beyond the lesion center into adjacent white matter and 28% (s.d. ± 7) extended 1.5 mm beyond the lesion site. Even at the relatively short 8-day time point, small numbers of axons extended still further, with averages of seven BDA^+ ^axons (s.d. ± 5; 7%) detected per animal at 5 mm rostral to the injury site and four axons (s.d. ± 3; 4%) in the dorsal column nuclei in GDA-transplanted animals. In contrast, in four out of five control animals, no axons were observed within the lesion centers or within white matter beyond the lesion. In just one out of five control animals, six BDA^+ ^axons (4%) were found in the ventral-most regions of the lesion site (that is, at the ventral margin), effectively rostral to the caudal lesion margin and therefore aligned with the lesion center. These were most likely to be due to a limited axonal sparing and/or sprouting in this animal, resulting in the presence of these axons in the ventral white matter of the cuneate pathway at the interface with gray matter. The fact that no BDA^+ ^axons were observed beyond 1.5 mm rostral to the lesion in this animal (Figure 2), or were observed crossing the injury site near the pial surface or within GFAP-negative regions of the lesion center proper in all control animals, supports the hypothesis that these six axons had sprouted around the injury at the gray/white matter interface rather than having been spared. Overall, approximately 99% of the cut ends of BDA^+ ^axons in control cords remained within caudal lesion margins and had dystrophic endings (Figure 3c). In sharp contrast, very few dystrophic axons were observed at the caudal interface of GDA transplants with adjacent white matter compared with control injury sites (compare Figure 3c and 3d).

### GDA 'bridge' supports axon growth

**Figure 4:**
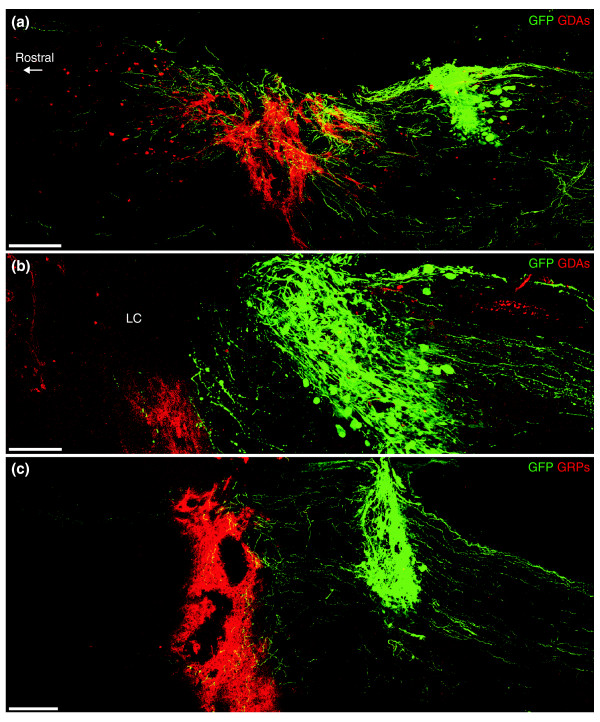
A comparison of the ability of GDA versus GRP transplants to promote axon growth across dorsal column injuries from adjacent microtransplanted adult sensory neurons at 8 days after injury and transplantation. **(a) **A montaged, confocal image scanned from a single 75-μm thick sagittally oriented section showing GFP^+ ^axons (green) entering and exiting a dorsal column lesion bridged with hPAP^+^(red) GDAs. **(b) **In two cases in which GDA transplants did not adequately fill the injury site or migrate into lesion margins, GFP^+^sensory axons failed to cross the caudal lesion margin and instead formed dystrophic endings identical to those in control untreated injuries. LC, lesion center. **(c) **Confocal montage showing the complete failure of transplanted GRPs to support the growth of GFP axons across a dorsal column injury. Note that, despite the ability of transplanted GRPs to span the injury site, the majority of GFP^+ ^axons have formed dystrophic endings within the caudal lesion margin. Scale bars: (a) 300 μm; (b) 100 μm; (c) 200 μm.

To further demonstrate the capacity of transplanted GDAs to support axon growth in an adult rat model of spinal cord injury that eliminates the possibility of axon sparing, we examined the ability of axons growing from adjacent transplants of adult DRG sensory neurons to cross identical spinal cord stab injuries bridged with GDAs. In these experiments, immediately after injury rats received microtransplants of adult mouse sensory neurons expressing green fluorescent protein (GFP) within dorsal column white matter 400–500 μm caudal to GDA-transplanted stab injuries (Figures 1c and 4a) or control stab injuries injected with media alone. In these experiments we also examined the ability of transplantation of GRP cells themselves to promote regeneration (Figures 1c and 4c).

**Figure 5:**
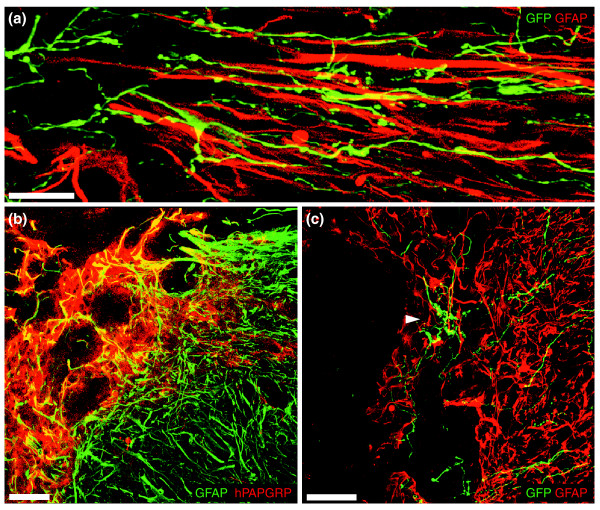
A comparison of GFP^+ ^axon and host astrocyte alignment in GDA- versus GRP- transplanted lesion margins at 8 days after injury. **(a) **A high-magnification image showing aligned axon growth (green) associated with aligned GFAP^+ ^host astrocytic processes (red) in the caudal margin of a GDA-transplanted lesion. **(b) **In contrast, GFAP^+ ^astrocytic processes (green) are misaligned in the caudal margin of a GRP-transplanted lesion (red). **(c) **A high-power confocal image showing GFP^+ ^axons displaying tortuous, misaligned patterns of growth and dystrophic end bulbs (arrowhead) within the astrogliotic caudal margin of a GRP-transplanted lesion. Scale bars: (a) 25 μm; (b,c) 50 μm.

Newly growing axons from the transplanted neurons failed to cross GRP-transplanted injuries (Figures 4c and 5c) or lesions injected with medium (data not shown). In contrast, 53% (s.d. ± 3) of rostrally directed GFP^+ ^axons grew into the center of GDA-transplanted injuries, 62% of axons at the lesion center reached 0.5 mm beyond lesion sites, 42% reached 1.5 mm into rostral white matter, and small numbers of axons extended up to 2 mm beyond the injury site (Figure 4a). Comparison of endogenous BDA^+ ^and GFP^+ ^axons from the two separate experiments (Table 1, experiments 1 and 2) revealed a remarkably similar efficiency of axon growth (66% and 62%, respectively) exiting GDA-filled injuries. Thus, transplantation of GDAs was able to promote axon growth across acute dorsal column injuries, but transplantation of GRP cells (from which GDAs are derived) had no such effect.

There was a striking correlation between the extent of axonal growth and the degree of occupancy (bridging) of the lesion by GDAs. In two GDA-transplanted animals in which GDAs did not completely fill the lesion site, very few GFP^+ ^axons penetrated the GDA-poor caudal lesion margins and GFP^+ ^axons within lesion centers were confined to areas containing GDAs (Figure 4b). In areas of the lesions devoid of GDAs, GFP^+ ^axons formed dystrophic endings within caudal lesion margins (Figure 4b). In these cases, no axons were observed to cross the site of injury and enter rostral white matter. GFP^+ ^axon growth was not fasciculated and was often aligned with human placental alkaline phosphatase (hPAP)-positive processes of GDAs and parallel with the host GFAP^+ ^astrocyte processes in the rostral and caudal lesion margins (Figure 5a). Similarly, BDA^+ ^endogenous axons were often aligned with hPAP^+ ^GDAs within rostral (Figure 3b) and caudal (Figure 3d) lesion margins.

As variation in lesion size has previously been observed after spinal cord injuries in different strains of adult mice [46], we conducted a qualitative assessment of lesion size and shape resulting from stab injuries of the dorsal columns in both Fischer 344 and Sprague Dawley rats, which revealed a degree of variability in Fischer 344 rats that was not observed in Sprague Dawleys (data not shown). This variation in lesion size and shape between individual Fischer 344 rats therefore precluded their use in quantitative tracing studies of endogenous axon regeneration (see Materials and methods section for further details). Both rat strains, however, showed equally consistent failure of GFP^+ ^axons to cross control lesions and both strains also showed successful axon growth across dorsal column injuries bridged with GDAs. Thus, treatment of dorsal column lesions with GDAs in two different strains of rat resulted in robust axon growth across sites of injury and failure of axons to traverse control injuries.

### Alignment of host tissue

**Figure 6:**
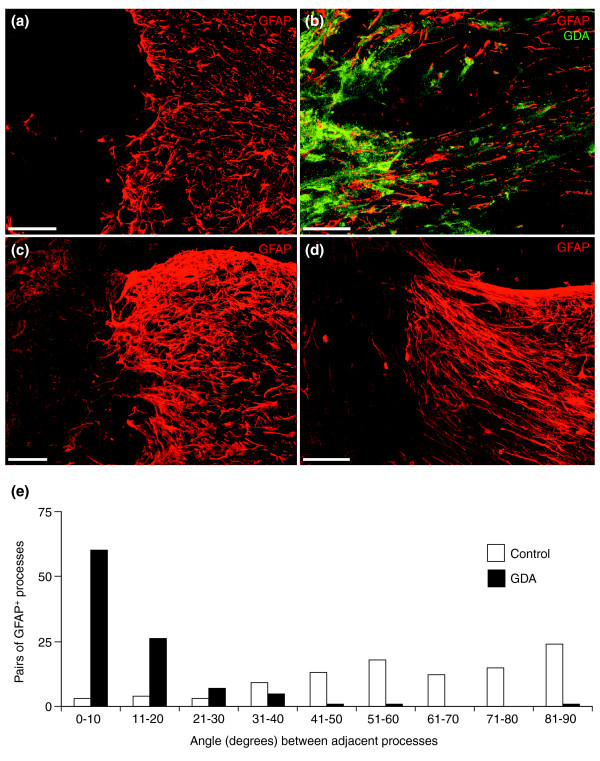
Reorganization of lesion margins by GDAs. **(a,c) **Control lesions; **(b,d) **transplanted lesions. Control lesions at (a) 4 days and particularly at (c) 8 days after injury have a dense meshwork of hypertrophic cell bodies and processes of endogenous astrocytes within lesion margins that is typical of forming glial scar tissue. (b) At 4 days after injury and transplantation, 'flares' of hPAP^+ ^GDAs (green) are interwoven with realigned host GFAP^+ ^astrocytes within lesion margins (the caudal margin is shown). Processes of both transplanted GDAs and host astrocytes are oriented towards the lesion center. Note that hPAP^+ ^GDAs are not GFAP^+^. (d) At 8 days after injury and transplantation, GDAs have effected a reduction in host astrogliosis and a striking realignment of host GFAP^+ ^astrocytes compared with the control (c). **(e) **Quantification of the alignment of host GFAP^+ ^processes in lesion margins. The angles measured between each pair of GFAP^+ ^processes in control (*n *= 100) and GDA-transplanted lesion margins (*n *= 100) are graphically displayed in a histogram. Each bin along the *x*-axis represents the angle between a pair of processes: 0° is parallel and 90° is perpendicular. The *y*-axis indicates the number of pairs of GFAP^+ ^processes within each bin. Note the striking difference in alignment of GFAP^+ ^host astrocytic processes in margins of GDA-transplanted lesions versus controls. GDA-transplanted lesions have an average angle of just 11.6° (median 7°) between paired processes, versus 59.4° (median 61°) for control lesion margins. Statistical analysis: *p *< 0.0001, *t*-test. Scale bars: (a,c,d) 100 μm; (b) 50 μm.

**Figure 7:**
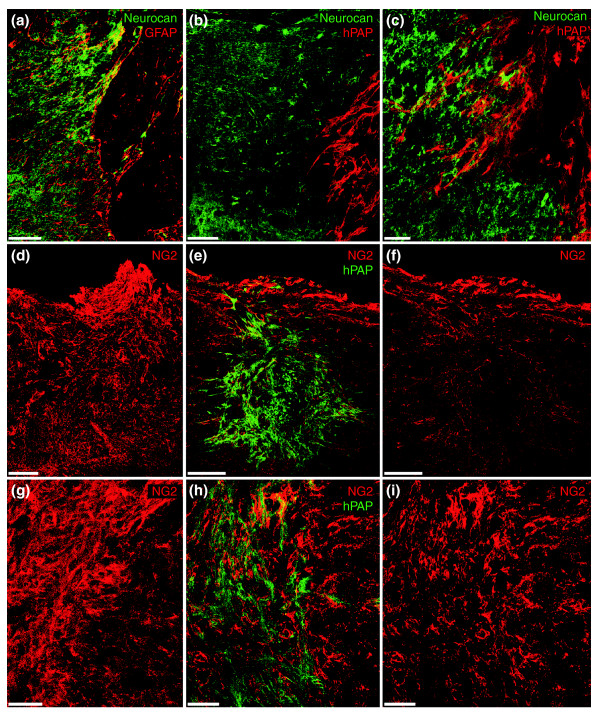
GDA transplantation suppresses neurocan and NG2 immunoreactivity. **(a) **At 4 days after injury, control lesion margins display dense neurocan immunoreactivity (green) mainly associated with fine, GFAP^- ^processes and to a lesser extent with GFAP^+ ^astrocyte cell bodies (red). **(b) **Neurocan immunoreactivity at 4 days after injury and transplantation is greatly reduced in margins of hPAP^+ ^GDA-transplanted lesions. **(c) **At 8 days after injury and GDA transplantation, neurocan immunoreactivity within lesion margins has increased compared with the 4-day time point. Note, however, that intra-lesion hPAP^+ ^GDAs continue not to be immunoreactive to neurocan. **(d-f) **GDA-transplanted lesion centers (e,f) at 4 days after injury show a marked reduction in NG2 immunoreactivity (red) compared with (d) control lesions. hPAP^+ ^cells are stained green. **(g-i) **Although overall NG2 immunoreactivity has increased within the center of GDA-transplanted lesions (h,i) at 8 days after injury compared with (e,f) the 4-day time point, it is reduced compared with the more uniformly distributed NG2 immunoreactivity within the center of control lesions at 8 days after injury. Scale bars: (a,b,g) 100 μm; (c,h,i) 50 μm; (d-f) 200 μm.

In both dorsal column axon regeneration experiments, the linearity of axonal growth we observed, particularly within lesion margins (Figures 3c,d and 5a), prompted us to examine the underlying tissue organization. Transplantation of dissociated GDAs was associated not only with a significant reduction in astrogliosis but also with a striking reorganization of host astrocyte cell bodies and processes within lesion margins (Figures 5a and 6b,d and Additional data file 1). To examine host astrocytes, we took advantage of an unexpected downregulation of GFAP in the transplanted GDAs at 4 and 8 days after transplantation (Figure 6b) to identify host astrocytes with anti-GFAP immunostaining. Intra-lesion GDAs did, however, remain positive for the astrocyte lineage markers S100 and vimentin (Additional data file 2) and did not express the oligodendrocyte lineage antigens NG2 (Figure 7e,h) or proteolipid protein (data not shown). GFAP^+ ^host astrocytes within the margins of control medium-injected lesions (Figures 3c, 6a,c and Additional data file 3), and in animals receiving GRP cell transplants (Figure 5b,c) exhibited the characteristic hypertrophic cell bodies of adult reactive astrocytes and formed a dense mass of numerous, ramified, misaligned processes typical of astrogliotic scar tissue. In contrast, in animals receiving GDA transplants, host GFAP^+ ^astrocyte processes within lesion margins were now oriented toward lesion centers (Figures 5a and 6b,d and Additional data file 1).

Quantitative analysis of the alignment of host GFAP^+ ^astrocytic processes in the lesion margins revealed considerable differences between GDA-transplanted and control injury sites. Control lesion margins had an average angle of 59.4° (s.d. ± 22, median = 61°) between adjacent pairs of astrocytic processes. In contrast, GDA-filled lesions had average angles of only 11.6° (s.d. ± 12.6, median = 7°) between adjacent host GFAP^+ ^processes within lesion margins (Figure 6e). Moreover, GDAs within lesion margins often interweaved with endogenous GFAP^+ ^astrocytes (Figure 6b), creating an aligned environment of glial cell surfaces, thus providing a directional guidance of axon growth across the interfaces of GDA-bridged lesions and adjacent white matter (Figures 5a and 6b,d and Additional data file 1).

### Suppression of inhibitory proteoglycans

GDA transplantation was also associated with a delayed expression of axon-growth-inhibitory proteoglycans in dorsal column lesions. The margins of control dorsal column lesions examined 4 days after injury displayed a high density of neurocan immunoreactivity associated with numerous, fine, GFAP-negative processes (Figure 7a), which we have previously shown to be primarily associated with NG2^+ ^glia [4]. In addition, NG2 immunoreactivity in control lesions was predominantly associated with invading meningeal fibroblasts and blood vessels in the center of control lesions (Figure 7d,g; see also [4]). In contrast, the margins of lesions containing GDA grafts at 4 days after injury showed a marked reduction in overall neurocan immunoreactivity (Figure 7b versus 7a), resembling instead the pattern of neurocan expression previously observed 2 days after injury in control lesions [4]. GDA-transplanted injury sites also showed reduced NG2 immunoreactivity compared with controls at 4 days after injury (Figure 7e,f). At the 8-day time point, however, neurocan immunoreactivity in the margins of GDA-transplanted lesions was similar in intensity and distribution to neurocan detected in control lesions at 8 days after injury (Figure 7c), indicating that the effect of the GDA transplant was to delay the expression of neurocan in lesion margins. Significantly, however, even at the 8-day time point GDAs within lesion margins and centers displayed little or no neurocan immunoreactivity (Figure 7c). Unlike the more uniform density of NG2 immunoreactivity within lesion centers at 8 days in control cords, NG2 immunostaining within GDA-transplanted injuries had a more patchy distribution (Figure 7h,i). This almost certainly reflected a chimeric mix of NG2^- ^GDAs with host NG2^+ ^tissue at lesion centers (compare Figure 7g and h,i), as we did not find hPAP^+^NG2^+ ^cells in the lesions even 8 days after transplantation.

### GDA transplantation promotes rubrospinal axon regeneration and suppression of red nucleus neuron atrophy

**Figure 8:**
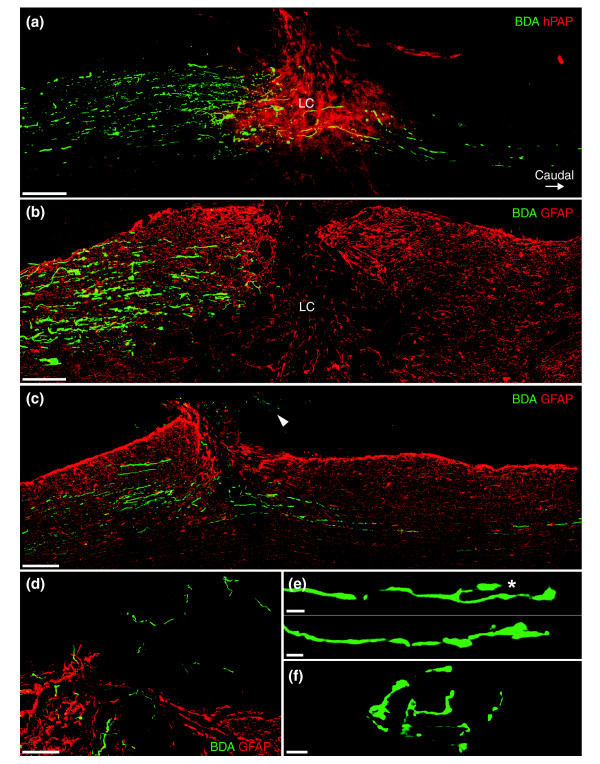
Transplanted GDAs promote regeneration of rubrospinal axons. **(a) **Confocal montage scanned through a depth of 60 μm, showing a small population of BDA^+ ^rubrospinal tract (RST) axons (green) that have traversed a GDA-bridged (red) lesion of the dorsolateral funiculus and entered caudal white matter at 8 days after injury. The majority of RST axons, however, have sprouted to within 300 μm of the lesion center (LC) but failed to extend beyond the site of injury. Note the absence of BDA-labeled axons within the dorsal-most regions of the injury site. **(b) **Confocal montage showing the complete failure of axotomized BDA^+ ^RST axons to cross control lesions at 8 days after injury and that the majority of axons have remained within rostral lesion margins at a distance of 500–800 μm from the lesion center (LC). **(c) **At 5 weeks after injury and transplantation, a small population of BDA^+ ^RST axons have traversed GDA-bridged injury sites and extended within caudal white matter. Note that BDA^+ ^axons have also sprouted into the dorsal regions of the lesion center and even extended beyond the pial surface (arrowhead; see also the high-power image in **(d)**). Note the lower levels of GFAP immunoreactivity (red) in more ventral regions of the injury margins and center, coincident with the presence of BDA^+ ^axons. **(e) **Two examples of RST axons displaying growth cones within white matter 2 mm caudal to a GDA-treated lesion, at 5 weeks after transplantation. Note the collateral branch (asterisk). **(f) **Confocal image of a BDA^+ ^terminal field-like axonal plexus within layer 5 spinal cord gray matter, immediately adjacent to the dorsolateral funiculus white matter at 5 weeks after injury and transplantation. In contrast, in all GDA-transplanted rats and controls injected with medium alone at 8 days after injury, no BDA labeling was observed within gray matter beyond the injury site. Scale bars: (a-c) 200 μm; (d) 100 μm; (e) 5 μm; (f) 10 μm.

GDA transplantation was also beneficial for CNS neurons, as demonstrated by analysis of rubrospinal tract (RST) axons within injuries to the right-side dorsolateral funiculus of the spinal cord and their corresponding neuronal cell bodies within the left-side red nucleus of the brain (Figure 1d). Severe injury to this descending, somatic motor control pathway disrupts the ability of rats to step rhythmically and coordinate accurate fore- and hind-limb placement. In animals in which the dorsolateral funiculus was transected, GDAs again filled the site of injury, integrated into host tissue and realigned host astrocytes. In animals receiving no GDAs, there was a complete absence of RST axons within the lesion centers (Figure 8b). The majority of BDA^+ ^axons in control injury sites had dystrophic endings and remained between 500 and 800 μm from lesion centers (Figure 8b). In sharp contrast, in four out of six animals receiving GDA transplants, BDA-labeled RST axons were readily observable within lesion centers (Figure 8a) and also within caudal white matter up to 1.5 mm beyond the site of injury. In addition, the majority of axotomized RST axons within GDA-transplanted animals were observed interacting with GDAs in rostral lesion margins and had sprouted to within 300 μm of lesion centers (Figure 8a). Those axons that had grown into caudal white matter in GDA-bridged injuries were invariably observed in the ventral half of the injury sites, which correlated with regions of GDA transplants that more often continuously spanned the injury site (Figure 8a). In the two out of six GDA-recipient animals in which GDA grafts did not span sites of injury (see also Figure 4b), no BDA axons were observed within white matter beyond the site of injury (data not shown).

We also examined the effects of GDA transplants at longer time points associated with ongoing behavioral recovery (as discussed later) and found that a transient presence of GDAs was apparently sufficient to have significant prolonged effects. At 5 weeks after injury, hPAP^+ ^GDAs were no longer detectable but BDA^+ ^RST axons were still observed within lesion centers and had extended further within caudal white matter up to 3 mm beyond sites of injury. Notably, in six of nine rats, RST axons were also now observed to have sprouted up within the more dorsal regions of lesion centers and to have even grown out into dorsal roots (Figure 8c,d). The presence of growth cones within caudal white matter (Figure 8e) clearly demonstrates that successful GDA transplants had stimulated RST axon regeneration beyond sites of injury. In two out of nine rats, a few widely dispersed axon arborizations, similar in morphology to those seen for axons innervating terminal fields, were also now detected in gray matter at distances of 1–2 mm beyond the injury site (Figure 8f). Ventral margins of GDA-transplanted injuries, which at earlier time points showed more effective spanning of the lesion, continued to show a rostro-caudal alignment of host astrocyte processes, thus demonstrating that the tissue alignment associated with GDA transplantation did not require the continued presence of GDAs for maintenance of this effect. The probable importance of GDAs in this effect was indicated by observations that rostro-caudal alignment of host astrocytes was less pronounced within dorsal lesion margins where GDA colonization was less complete at earlier time points. The dorsal host astrocytes also appeared more hypertrophic than the ventral host astrocytes (Figure 8c). Nonetheless, even dorsally, host astrocytes were more aligned and less reactive than in control animals. In contrast to the beneficial effects associated with GDA transplantation, in control rats that received lesions and cyclosporine but no GDA transplants the RST axons remained in the rostral lesion margins at 5 weeks after injury and had dystrophic endings (data not shown).

**Figure 9:**
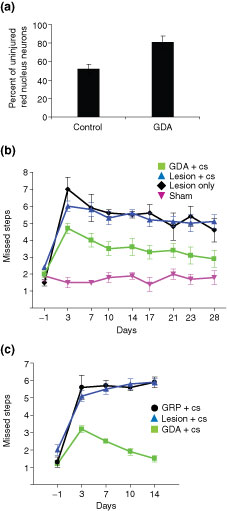
GDA transplantation suppresses atrophy of red nucleus neurons and promotes robust behavioral recovery. **(a) **Injured left-side red nuclei in control rats contained an average of 52% of the neurons counted in uninjured right-side red nuclei at 5 weeks after injury. The numbers of neurons in the injured left-side red nuclei of GDA-transplanted animals, however, was 81% of total neuron numbers in uninjured right-side nuclei (*p < 0.01). **(b) **Grid-walk analysis of locomotor recovery. Graph showing the average number of mistakes per experimental group at different time points after injury for GDA-transplanted rats versus the control-lesion and sham-operated groups. GDA-transplanted animals (green) performed significantly better than lesioned controls at all post-injury time points (*p *< 0.05). **(c) **Transplanted GRPs do not promote locomotor recovery. Graph showing the average number of grid-walk mistakes per experimental group from 1 day before injury (baseline pre-lesion) to 2 weeks after injury for a separate series of matched RST-lesioned rats that received either GRP or GDA transplants versus lesion-only control rats. Note the complete failure of locomotor recovery in GRP-transplanted animals compared with lesion-only controls at all time points and confirmation of significant locomotor recovery in response to GDA transplantation (*p *< 0.05). cs, cyclosporine.

GDA transplants to dorsal lateral funiculus lesions also resulted in a suppression of atrophy of neurons within the injured red nucleus (Figure 9a and Additional data file 4). Atrophy of significant numbers of red nucleus neurons begins 1 week after RST transection [47]. We similarly found that the number of neurons with a cell body diameter greater than 20 μm in the injured left-side red nucleus in rats not receiving GDA transplants fell to 52% of the values in the uninjured right-side nucleus at 5 weeks after injury. Design-based stereological analysis (see Materials and methods) revealed, however, that the injured left-side nucleus in GDA-transplanted animals contained 81% as many large-diameter neurons as found in the uninjured right-side nucleus, effectively an approximately 65% increase in numbers of neurons that had maintained a cell body diameter of greater than 20 μm above that observed for control, injured red nuclei (Figure 9a).

### GDA transplantation promotes behavioral recovery

A further indication of the efficacy of GDAs in promoting CNS recovery was seen behaviorally. Transection of the dorsolateral funiculus severs descending, supraspinal axons and results in chronic deficits in both fore- and hind-limb motor function [48], which can be detected by the grid-walk behavioral test [49]. Following transection of the dorsolateral funiculus, rats that received GDA transplants performed significantly better than controls at all post-surgery time points (Figure 9b) and their behavior improved significantly between 3 days and 28 days after injury (two-way repeated measures ANOVA, *p *< 0.05). Rats that received GDA transplants made an average of 4.7 mistakes at 3 days after injury and transplantation and improved to an average of 2.9 mistakes at 28 days after injury. In contrast, control lesioned rats made on average 6.0 mistakes at 3 days after injury, and showed no statistically significant improvement at any later time point, with an average of 5.1 mistakes at 28 days after injury. Before surgery, rats in control and treated groups performed equally well, with a baseline average of 2.0 mistakes. Thus, the average number of mistakes made by GDA-transplanted animals had improved to just 0.9 points above baseline, compared with no recovery of control lesioned rats at 28 days after injury. Moreover, analysis of individual rats at 28 days showed that four out of nine lesioned animals that received GDA transplants had scores that were now statistically identical to their pre-surgery baseline scores (data not shown). As discussed later, it thus appears that GDA transplantation was associated both with reductions in the extent of neurological deficit at 3 days after injury and with further recovery in the 4 weeks following injury.

Our data clearly demonstrate the failure of transplanted GRP cells to suppress scar formation and support axon growth across acute spinal cord injuries. In the light of a recent study showing the ability of GRPs to suppress glutamate-mediated neurotoxicity *in vitro *[50], there remained, however, the potential for acutely transplanted GRPs still to promote functional recovery via this mechanism, an effect that would be detectable during the first week after injury. To investigate this hypothesis, we conducted an analysis of grid-walk performance at time points ranging from 3 days to 2 weeks after injury in a further series of matched RST-lesioned rats that received transplants of GRP cells, GDAs or control medium injections. In accordance with previous results, GDA-transplanted rats once again showed a significant recovery of locomotor function compared with controls (Figure 9c) at all time points after injury (two-way repeated measures ANOVA, *p *< 0.05). Notably, at 14 days after injury, GDA-transplanted rats had an average score of 1.5 (± 0.2) mistakes compared with 5.9 (± 0.2) mistakes for lesion-only controls (Figure 9c). In sharp contrast, GRP-transplanted animals showed no recovery of locomotor function compared with controls at all time points after injury (Figure 9c).

## Discussion

We have demonstrated that astrocytes derived from embryonic spinal cord GRP cells can promote axon regeneration and functional recovery after transplantation into acute adult spinal cord injuries. The ability of GDAs to fill the injury site, suppress astrogliosis, realign host tissues and delay expression of axon-growth-inhibitory proteoglycans suggests that these cells are unusually effective in providing an environment that supports axon growth within acute CNS injuries. These attributes, in combination with their ability to reduce atrophy of axotomized CNS neurons and promote a significant behavioral recovery, make GDAs an attractive novel cell type with which to repair the damaged CNS.

The effects of GDAs on the growth of both ascending dorsal column axons and descending RST axons beyond sites of injury compare favorably with previous transplant-based spinal cord injury therapies. Intra-lesion sciatic nerve grafts have proven to be relatively poorly supportive of sensory axon reentry into white matter rostral to dorsal column transection injuries without additional treatments that support axon growth [51]. Intra-lesion transplantation of marrow stromal cells alone has not resulted in any sensory axons exiting the rostral margins of grafts [52] and, although the ability of regenerating sensory axons to cross dorsal column or dorsal root entry zone injuries bridged by olfactory ensheathing cells is still contentious [53-56], it is generally accepted that intra-lesion Schwann cell and neonatal astrocyte grafts are also poorly supportive of axon reentry into host white matter [33,57]. Although the growth of RST axons into and beyond GDA-bridged lesions was less efficient than that observed for ascending axons of the dorsal columns, the ability of RST axons to exit GDA-bridged lesions and extend up to 3 mm beyond the injury nonetheless offers a marked improvement over the complete failure of RST axons to cross spinal cord injuries bridged with olfactory ensheathing cells [58].

Our experiments demonstrate the value of pre-differentiation of precursor cells prior to transplantation and show that the signals encountered within the lesion site are not able to convert GRP cells themselves to a population that supports axon growth. This was confirmed by the complete failure of transplanted GRP cells to suppress scar formation and provide a bridge that supports axon growth. The fact that pre-differentiation of these cells to GDAs is required for repair of acute spinal cord injuries was also confirmed by the inability of transplanted GRP cells to support locomotor recovery in our RST-lesion model. These data are consistent with previous studies showing a failure of undifferentiated GRP cells to promote supraspinal axon regeneration [24] or behavioral recovery [19] after transplantation to spinal cord injuries. Whether this is because the adult lesion environment lacks the signaling molecules required to generate GDA-like cells from transplanted GRP cells *in vivo *or whether those signals are overridden by other influences, such as inflammatory cytokines, remains to be investigated.

Determination of the exact mechanisms by which any cell type provides benefit after transplantation to the traumatically injured CNS is challenging given the wide range of possible effects of such procedures, and there are a variety of means by which GDA transplantation could have contributed to the significant recovery of locomotion we observed in our grid-walk experiments. The early onset of recovery at the 3-day time point suggests an initial neuroprotective effect associated with GDA transplantation, consistent with our observed rescue of red nucleus neurons from atrophy. Rescue of red nucleus neurons from atrophy has been achieved through provision of brain-derived neurotrophic factor (BDNF [59]). Ongoing analyses of gene expression in GDAs shows readily detectable levels of BDNF mRNA (C.P., unpublished observations). In contrast, previous studies indicate that postnatal type-1 astrocytes (derived from the cortex) do not make BDNF [42], revealing another advantage of GDAs. Thus, it is apparent that the antigenic category of type-1 astrocytes [60] in which GDAs were originally placed [35,36] is too broadly defined and needs refinement.

The significant increases in behavioral recovery from 3 days onwards observed in GDA-treated animals in two separate experiments also suggests that axon regeneration and/or plasticity of connection may have contributed to overall functional recovery. As suppression of atrophy of axotomized red nucleus neurons has also been associated with regeneration of their axons into sciatic nerve grafts [59], the significantly greater number of neurons with cell body diameters over 20 μm in the injured red nucleus of GDA-treated animals at 5 weeks after injury, combined with the further elongation of RST axons in caudal white matter observed between 8 days and 5 weeks, supports a possible contribution of RST axon growth and plasticity to overall behavioral recovery. The relatively modest extent of RST axon growth, however, and the formation of terminal-like structures within adjacent gray matter at the spinal level of cervical vertebra C4, makes it likely that effects of GDAs on other pathways also contributed to functional recovery. Previous studies have shown that recovery of locomotor function after dorsal spinal cord hemisection injuries is associated with plasticity of corticospinal innervation of surviving propriospinal pathways, which in turn form new connections with denervated motor neurons [61]. The ability of GDAs to provide benefit to both ascending dorsal column and RST axons raises the possibility that GDAs will also be found to support the recovery of other axon populations relevant to locomotion, such as those in the descending reticulospinal and lateral corticospinal pathways [49].

We have previously shown that decorin-mediated suppression of the levels of the core proteins and glycosaminoglycan side chains of CSPGs can render spinal cord injuries more permissive for axon growth [62]. The delayed expression of inhibitory CSPGs associated with GDA transplantation therefore seems likely to have had an important role in enabling regenerative axon growth. The absence of neurocan and NG2 immunoreactivity shown by GDAs within sites of injury indicates that intra-lesion GDAs may be refractory to signaling molecules known to induce expression of neurocan in neonatal astrocyte cultures [63]. Thus, intra-lesion GDAs maintained an axon-growth-supportive phenotype with respect to CSPG expression. Moreover, the presence of GDAs within lesions also modified the host response to injury and resulted in a significant reduction in NG2 expression within lesion centers and a delay in neurocan expression at lesion margins, which together may have created a window of opportunity for axons not only to enter but also to exit the injury site. In this context, the greater extent of initial RST axon retraction from the injury site compared with ascending dorsal column axons may mean that all but the fastest-responding RST axons missed this window of inhibitor suppression. The ability of decorin infusion to maintain a significant reduction in the levels of multiple axon-growth-inhibitory CSPGs within acute spinal cord injuries at 8 days after injury [62] suggests that a combined treatment with GDA and decorin may extend the window of opportunity for acute RST axon regeneration.

Also of potential importance to the ability of GDAs to promote axonal regeneration, and perhaps one of the most interesting effects of GDA transplantation, was the extent of linear tissue organization induced by these cells at sites of injury. Although previous studies have demonstrated that alignment of host astrocytic processes alone is not sufficient to promote axon growth across CNS injuries in the presence of inhibitory CSPGs [12,64], clearly the efficiency of axon growth across an injury site with reduced inhibitor expression will be enhanced if axons are not required to negotiate a maze of misaligned cellular processes, that is, if they can take the shortest route. The fact that a dissociated suspension of GDAs is able to effect a linear alignment within acute adult spinal cord injuries without the addition of an aligned bio-matrix suggests that the creation of such tissue organization is a fundamental aspect of the biology of these cells.

## Conclusion

In summary, GDA transplantation into the injured spinal cord promoted levels of axon regeneration, alignment of host tissue, suppression of scar formation, neuronal rescue and locomotor recovery that have not been associated with transplantation of other cell types. Critically, these benefits were dependent upon pre-differentiation of glial precursors to a desired astrocytic phenotype prior to transplantation and were not observed with transplantation of glial-restricted precursors. Our study demonstrates that the environment of acute, adult spinal cord injuries does not promote differentiation of glial precursors into the most advantageous cell type for tissue repair and functional recovery. Achieving such linear tissue organization, robust axonal growth and functional recovery in the absence of additional biomaterials, cell modification, or delivery of adjunctive bioactive therapies leads to great interest in now determining whether the beneficial effects of transplanted astrocytes derived from embryonic precursors can be enhanced still further by the application of rational combination therapies.

## Materials and methods

### Isolation of GRPs and generation of GDAs

GRPs labeled with A2B5 antibody (a cell-surface glycolipid unique to this cell type during early stages of rat spinal development) were isolated by fluorescence-activated cell sorting of dissociated cell suspensions from spinal cord of embryonic day 13.5 transgenic Fischer 344 rat embryos expressing the gene for hPAP under the control of ROSA26 promoter (transgenic rat line:TgN(R26ALPP)14EPS) [65]. GRPs were maintained in culture with DMEM-F12 media (Gibco/Invitrogen, Carlsbad, USA) with 10 ng/ml basic fibroblast growth factor (bFGF; Sigma, St. Louis, USA) and N2 tissue culture supplement (Gibco/Invitrogen) on a mixed laminin/fibronectin substrate and exposed to 10 ng/ml of human recombinant BMP-4 (R&D Systems) for 7 days in culture to differentiate them into A2B5-negative astrocytes. The following culture conditions were controlled and consistent between batches of GDAs: growth substrate, cell density, growth media, cell feeding schedule, the concentrations and source of growth factors, the total length of time in culture and the number of passages of GRPs before initiating the differentiation protocol.

### Characterization of the GDA phenotype *in vitro*

Cells in culture were labeled live with A2B5 monoclonal antibody (Chemicon, Temecula, USA; for GRPs or type-2 astrocytes) or anti-NG2 antibodies (Chemicon; for oligodendrocyte precursors), then fixed with cold acid and alcohol and labeled with antibodies for GFAP (Sigma; for astrocytes), FGF receptor 3 (Sigma; for type-1 astrocytes), or proteolipid protein (plp)/DM20 (Chemicon; for oligodendrocyte lineage cells). Secondary antibodies were purchased from Jackson Immunologicals (West Grove, USA) and Molecular Probes (Eugene, USA). BMP-4-induced GDAs were uniformly immunoreactive for human alkaline phosphatase *in vitro*. Although no NG2^+ ^or plp/DM20^+ ^oligodendrocyte precursors were detected in these cultures, undifferentiated GRPs (A2B5^+^GFAP^-^) or astrocytes of a type-2 phenotype (A2B5^+^GFAP^+^) were occasionally detected after BMP-4 treatment. These cell types represented less than 1% of the total cell population. To ensure GDA suspensions for transplantation did not contain undifferentiated GRPs or cells with the phenotype of type-2 astrocytes, potentially contaminating cell types were removed from the suspension by immuno-panning with the anti-A2B5 antibody. A small volume of the resulting suspension was plated onto glass coverslips and labeled with antibodies to A2B5 and GFAP to verify a uniform type-1 astrocyte phenotype. For transplantation, 100% GFAP^+^A2B5^- ^GDAs were suspended in Hanks Balanced Salt Solution (HBSS) at a density of 30,000 cells/μl.

### Lesion models and cell transplantation

Adult female Sprague Dawley or Fischer 344 rats (3 months old, Harlan, Indianapolis, USA) were anesthetized by injection of a cocktail containing ketamine (42.8 mg/ml), xylazine (8.2 mg/ml) and acepromazine (0.7 mg/ml). For dorsal column injuries (Figure 1a–c), the right-side dorsal column was unilaterally transected between cervical vertebrae 1 and 2 using a 30-gauge needle as a blade (see also [4,11,62]). Lesions extended to a depth of 1 mm and extended laterally 1 mm from the midline. For rubrospinal tract lesions, unilateral transections of the right-side dorso-lateral funiculus including the rubrospinal pathway were conducted at the C3/C4 spinal cord level with microscissors (Fine Science Tools, Foster City, USA). Lesions extended to a depth of 1 mm and extended medially 1 mm from the lateral pial surface of the spinal cord (Figure 1d).

A total of 4 μl of GDA or GRP suspension (30,000 cells/μl; 120,000 cells) per animal was acutely transplanted into six different sites in dorsal column lesions (two injections each into medial and lateral regions of the rostral and caudal lesion margins, and two injections into medial and lateral regions of the lesion center; Figure 1b). All dorsal column *in vivo *experiments were conducted in the absence of immunosuppressants. GDA or GRP transplants were injected in an identical pattern into lesions of the dorsolateral funiculus and a total of 6 μl of GDA or GRP suspension (30,000 cells/μl; 180,000 cells) injected per injury site. Control lesion rats were injected with 6 μl HBSS. One set of rats in the dorsolateral funiculus lesion-only group and all rats that received GDA or GRP transplants and dorsolateral funiculus lesions were administered daily injections of cyclosporine (1 mg per 100 g body weight) beginning the day before injury and transplantation through to experimental endpoints. Sham-operated rats in which the spinal cord was exposed but not lesioned, and rats that received a lesion but no cyclosporine, were included as control groups (Table 1).

Our previous studies have characterized scar formation and CSPG expression after spinal cord injury in adult Sprague Dawley rats, a strain commonly used in CNS regeneration studies. Unilateral dorsal column stab injuries identical to those in the present study reliably generated lesions of uniform size and induced consistent, quantifiable changes in CSPG expression in adult female Sprague Dawley rats [4,62]. As the GDAs were derived from hPAP Fischer 344 rats, however, we conducted an initial pilot series of intra-lesion GDA transplants versus untreated controls in dorsal column injuries of both adult female Fischer 344 and Sprague Dawley rats and assayed the ability of axons growing from adjacent DRG neuron transplants to cross sites of injury versus controls that did not receive GDAs. Although GFP^+ ^axons consistently failed to cross control lesions in both strains of rats, we observed significant variations in lesion size and margin morphology in control Fischer 344 rats, a phenomenon that we did not observe in Sprague Dawley rats. The greater variation in lesion size and rostro-caudal distances of lesion margins from lesion centers in Fischer 344 rats precluded accurate quantification of the numbers of endogenous, BDA-labeled axons at set distances from lesion centers in this strain of rats. Therefore, a separate study to investigate and quantify regeneration of BDA^+ ^endogenous ascending dorsal column axons and CSPG expression in GDA-transplanted versus control dorsal column lesion animals was conducted in Sprague Dawley rats (see Table 1 for the rat strains and numbers of animals used in each study). Thus, bridging dorsal column lesions with GDAs in two different strains of rat, in two separate axon regeneration experiments, both resulted in robust axon growth across sites of injury and failure of axons to traverse control injuries.

### Adult DRG neuron transplantation

Single-cell suspensions of adult mouse sensory neurons were prepared from 10- to 12-week-old transgenic mice expressing the gene for enhanced GFP [66] as previously described [11,12,62]. No growth factors were added to the neuron suspension. 500 nl of the neuron suspension (about 1,500 neurons/μl) was acutely microtransplanted into dorsal column white matter approximately 500 μm caudal to the lesion (Figure 1c).

### Histology

At 4 days, 8 days and 5 weeks after surgery, animals were deeply anesthetized and transcardially perfused with 0.1 M PBS followed by 4% paraformaldehyde in 0.1 M PBS. For frozen sectioning, dissected spinal cords were cryoprotected in a 30% sucrose/PBS solution at 4°C overnight. Tissue was embedded in OCT (Sakura Finetek, Torrance, USA) and quickly frozen. Serial 25-μm thick frozen sections were cut in the sagittal plane and air-dried onto gelatin-coated glass slides. For vibratome sectioning, dissected spinal cords were post-fixed in 4% paraformaldehyde overnight, then embedded in 5% gelatin/5% agar. Serial 75-μm thick sagittal sections were collected and processed as free-floating sections. All tissue sections were washed in PBS, blocked with 4% normal goat serum in solution with 0.1% triton/PBS for 30 min, then incubated with appropriate primary antibodies in the blocking solution overnight at 4°C. Secondary antibody incubations were for 1 h at room temperature.

The following primary antibodies were used: monoclonal anti-GFAP (Sigma) and polyclonal anti-GFAP (Sigma); monoclonal anti-vimentin (Chemicon); monoclonal anti-plp/DM20 (Chemicon); polyclonal anti-NG2 (Chemicon); polyclonal anti-GFP (Molecular Probes); monoclonal anti-hPAP (Sigma); polyclonal anti-hPAP (Fitzgerald, Concord, USA). Secondary antibodies conjugated with Cy5, Cy2 (Jackson), Alexa-488 or Alexa-594 (Molecular Probes) were used to visualize primary antibody binding. All secondary antibodies were pre-absorbed against rat serum. To control for nonspecific binding of secondary antibodies, adjacent sections were also processed as described above without primary antibodies. Labeled sections were examined using an Olympus BX60 fluorescence light microscope and a Leica TCS SP2 confocal microscope. Molecule co-localization and cellular associations were determined with Leica three-dimensional analysis software. All immunohistological images were acquired with confocal microscopy (Leica TCS SP2) of sections cut in the sagittal plane. Spinal cord rostral to the lesion is shown to the left in all figures.

### Tracing endogenous dorsal column or rubrospinal axons

In both lesion models, endogenous axons were traced by injection of 10% BDA in sterile PBS (Molecular Probes) 8 days before an experimental endpoint. In the dorsal column lesion model, ascending endogenous axons were traced by BDA injection to a depth of 0.5 mm into the right-side cuneate and gracile white matter at the C3/C4 spinal level (Figure 1c). Descending RST axons were traced in the dorsolateral-funiculus lesion model by injection of BDA into the magnocellular region of the left-side red nucleus (coordinates: 6.04 mm posterior, +0.7 mm lateral and 7.6 mm below bregma). For histological analysis of BDA-labeled axons, 25 μm serial sagittal sections were collected and processed for immunohistochemistry, as described above. BDA was visualized by incubating tissue sections with the VectastainABC solution (Vector Labs, Burlingame, USA), and further intensified with the Tyramide-Alexa 488 reagent (Molecular Probes).

### Quantification of endogenous ascending dorsal column axons

The number of BDA-labeled axons was counted in every third tissue section spanning the medial-lateral extent of dorsal column injury sites at the following locations: 0.5 mm caudal to the injury; directly at the injury center; 0.5 mm, 1.5 mm and 5 mm rostral to the injury site; and within the dorsal column nuclei. To control for differences in axon tracing and labeling efficiency between animals, the numbers of BDA-labeled axons counted within the lesion center and at all rostral sites were normalized to the number of BDA-labeled axons detected 0.5 mm caudal to the lesion site for each tissue section examined. The normalized values from each tissue section for each separate animal (control and GDA-transplanted) were averaged to generate values for each animal. The values for each animal (*n *= 6 GDA-transplanted, 5 control) were then averaged and displayed graphically. ANOVA or *t*-tests were performed as appropriate, with a value of *p *< 0.01 taken to be significant. For separate experiments analyzing the growth of GFP^+ ^axons from microtransplanted sensory neurons, identical methods were used to count GFP^+ ^axons from alternate sagittally orientated 75 μm vibratome sections.

### Quantification of alignment of host GFAP^+ ^astrocyte processes

Confocal images were generated from scanning through 30-μm thick sagittal oriented sections of caudal and ventral dorsal column lesion margins immunostained for GFAP to show host astrocytic processes. Five sections were selected from the lateral to medial center of lesions in three control and three GDA-transplanted rats (see Additional data files 1 and 3). Within each confocal image, GFAP^+ ^processes were randomly selected within the lesion margin and 'best fit' lines traced over them using Image Pro Plus software (Media Cybernetics, Silver Spring, USA). Then an immediately adjacent GFAP^+ ^process was identically traced and the angle between the lines calculated with the Image Pro Plus software. In all, 20 pairs of GFAP^+ ^host astrocytic processes from each confocal image (5 images per group) were analyzed and the mean and median angles were determined. A *t*-test was performed to determine the statistical significance of the difference in measured angles between astrocytic processes for GDA and control groups, with a highly significant *p *value of < 0.0001.

### Grid-walk behavioral analysis

Two weeks prior to surgery, rats were trained to walk across a horizontal ladder (foot misplacement apparatus, Columbus Instruments, Columbus, USA) and only rats that consistently crossed without stopping were selected for experiments. The grid-walk test is a sensitive measure of the ability of rats to step rhythmically and to coordinate accurate placement of both fore- and hind-limbs. For analysis of acute to long-term recovery of locomotor function in GDA-transplanted versus untreated lesion controls (Table 1, Experiment 3), trained rats were randomly assigned to one of four groups: RST lesion + GDA + cyclosporine (*n *= 9); RST lesion + suspension media + cyclosporine (*n *= 9); RST lesion only (*n *= 7); and sham operation (*n *= 7). One day before surgery (baseline) and at time points of 3, 7, 10, 14, 17, 21, 24, and 28 days after surgery, each rat was tested three times and the number of missteps from each trial was averaged to generate a daily score for each animal. To compare the effects of GRP and GDA transplants on recovery of locomotor function (Table 1, Experiment 4), a further series of trained rats were randomly assigned to one of three groups: RST lesion + GRPs + cyclosporine (*n *= 6); RST lesion + GDAs + cyclosporine (*n *= 5); and RST lesion + medium injection + cyclosporine (*n *= 6). Grid-walk performance was tested in an identical fashion to that used for rats in Experiment 3 at time points of 3, 7, 10, and 14 days after injury. Two-way repeated measures ANOVA and the Tukey post test (with a significance level of *p *< 0.05) were used to analyze both datasets.

### Quantification of red nucleus neurons

At 5 weeks after injury and transplantation, 25 μm serial frozen sections were cut in the coronal plane from the brains of rats that had undergone behavioral analysis. Every third section through the rostro-caudal extent of the red nucleus was stained with 0.2% cresyl violet. Standard, design-based stereology methods (CAST software, Olympus, Melville, USA) were used to quantify numbers of neurons in both red nuclei of RST-lesioned, GDA-transplanted (*n *= 6) and control (medium injection + cyclosporine, *n *= 6) rats. An optical fractionator was applied to left- and right-side red nuclei from every sixth section. Cell bodies greater than 20 μm in diameter and characteristic neuronal morphology were counted. The numbers of neurons counted in the left-side injured red nucleus were normalized to counts obtained for the uninjured right-side nucleus for each animal. The values for each animal within a group were averaged and displayed graphically. A *t*-test was performed to determine the statistical significance of the difference between the groups (with a value of *p *< 0.01 taken to be significant).

All procedures were performed under the guidelines of the National Institutes of Health and approved by the Institutional Animal Care and Utilization Committee of Baylor College of Medicine, Houston, USA.

## Additional data files

The following files are available: a figure showing the alignment of host GFAP^+ ^processes in animals that have received GDA transplants (Additional data file 1); a figure showing the expression of astrocytic markers by GDAs *in vivo *(Additional data file 2); a figure showing misaligned host astrocytic processes in control lesions (Additional data file 3); and a figure showing suppression of atrophy of axotomized red nucleus neurons by intra-spinal transplanted GDAs (Additional data file 4).

## Supplementary Material

Additional data file 1 A figure showing the alignment of host GFAP^+ ^processes in animals that have received GDA transplants Click here for additional data file

Additional data file 2 A figure showing the expression of astrocytic markers by GDAs *in vivo * Click here for additional data file

Additional data file 3 A figure showing misaligned host astrocytic processes in control lesions Click here for additional data file

Additional data file 4 A figure showing suppression of atrophy of axotomized red nucleus neurons by intra-spinal transplanted GDAs Click here for additional data file

## Figures and Tables

**Glossary:**
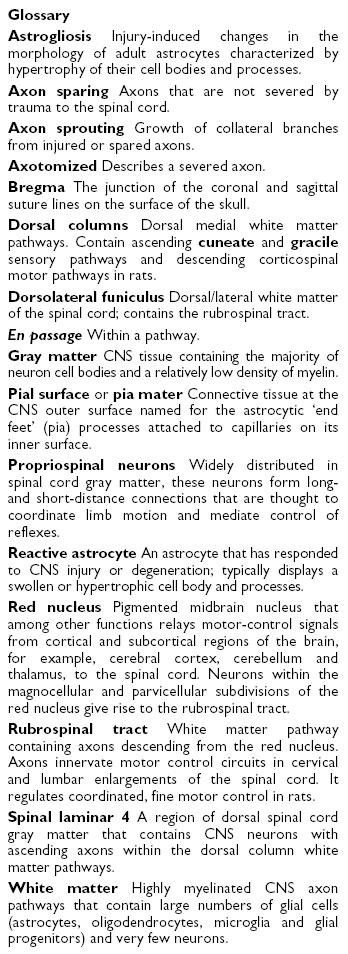
Glossary

**Table 1:** Numbers of animals per experimental group

Experiment	Details	Strain	Time point	Control lesion	+GDA	+GRP
1	Analysis of endogenous sensory axon regeneration, CSPG expression and GDA phenotype	Sprague Dawley	4 days	4	6	
			8 days	5	6	
						
2	Analysis of axon growth from GFP^+ ^transplanted sensory neurons	Fischer 344	8 days	6	9	
		Sprague Dawley	8 days	4	4	
			8 days	4		6
						
3	Analysis of RST axon growth, red nucleus and behavioral recovery	Sprague Dawley	8 days	6 + cyc	6 + cyc	
			5 weeks	9 + cyc	9 + cyc	
			5 weeks	7		
			5 weeks	7 (sham)		
						
4	Analysis of acute behavioral recovery	Sprague Dawley	14 days	6 + cyc	5 + cyc	6 + cyc

Cyc, cyclosporine
