# Supplementary material for: Astrocytes derived from glial-restricted precursors promote spinal cord repair
Source: J Biol. 2006 Apr 27;5(3):7. doi: 10.1186/jbiol35 (PMC1561531; doi:10.1186/jbiol35)
Supplement: Additional data file 1 — A figure showing the alignment of host GFAP+ processes in animals that have received GDA transplants [file jbiol35-s1.pdf]

Additional data file 1

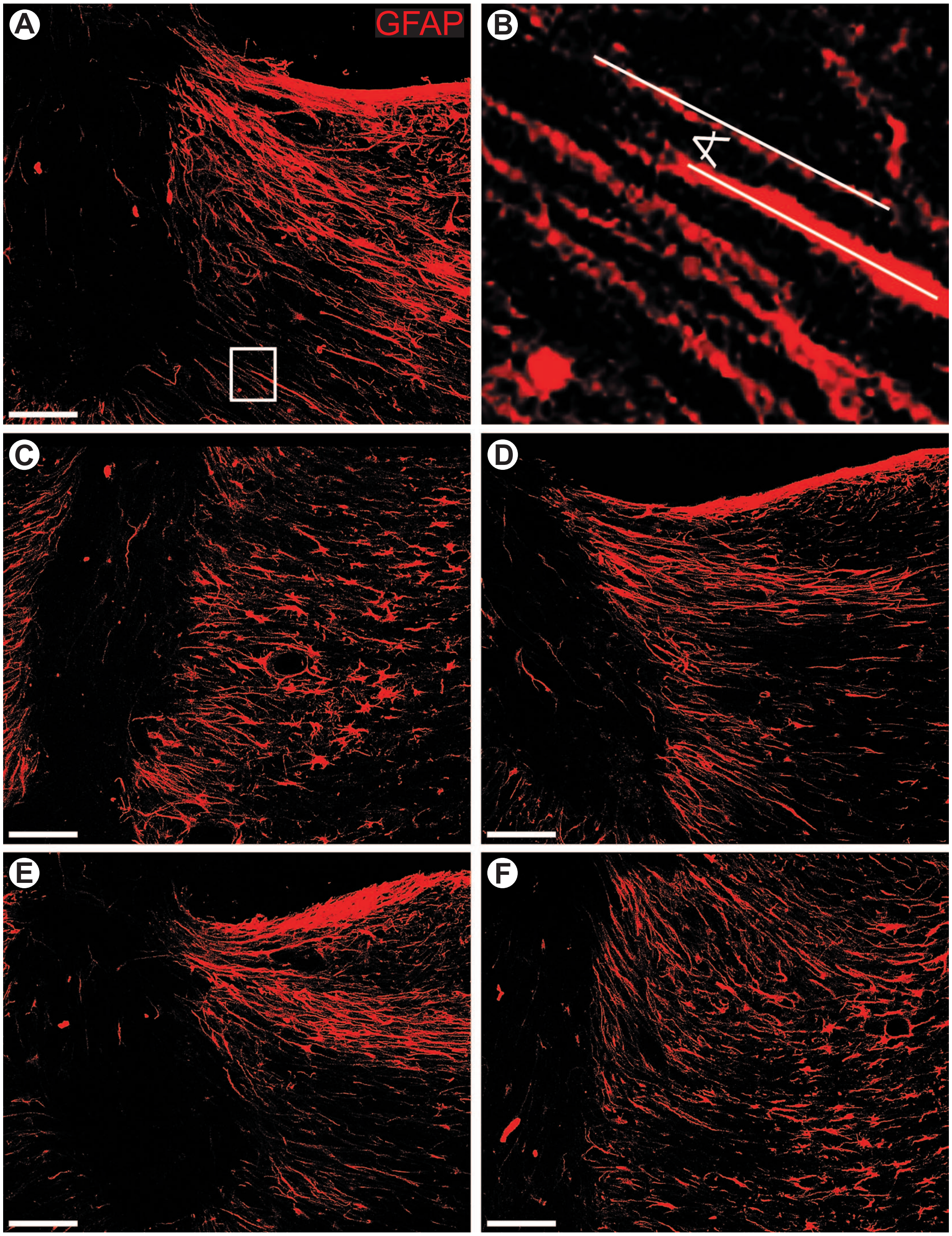

Additional figure 1

Alignment of host astrocytes. **(a,c-f)** Confocal images showing a striking alignment of host GFAP+ processes in caudal lesion margins at 8 days after injury in three animals that have received GDA transplants. **(b)** High-power image of the boxed area in (a). Average angles of only 11.6° (s.d. ± 12.6, median = 7°) were recorded between adjacent host GFAP+ processes within margins of GDA-transplanted injury sites. Scale bars in (a,c-f) represent 100 μm.
