# Supplementary material for: Astrocytes derived from glial-restricted precursors promote spinal cord repair
Source: J Biol. 2006 Apr 27;5(3):7. doi: 10.1186/jbiol35 (PMC1561531; doi:10.1186/jbiol35)
Supplement: Additional data file 2 — A figure showing the expression of astrocytic markers by GDAs in vivo [file jbiol35-s2.pdf]

Additional data file 2

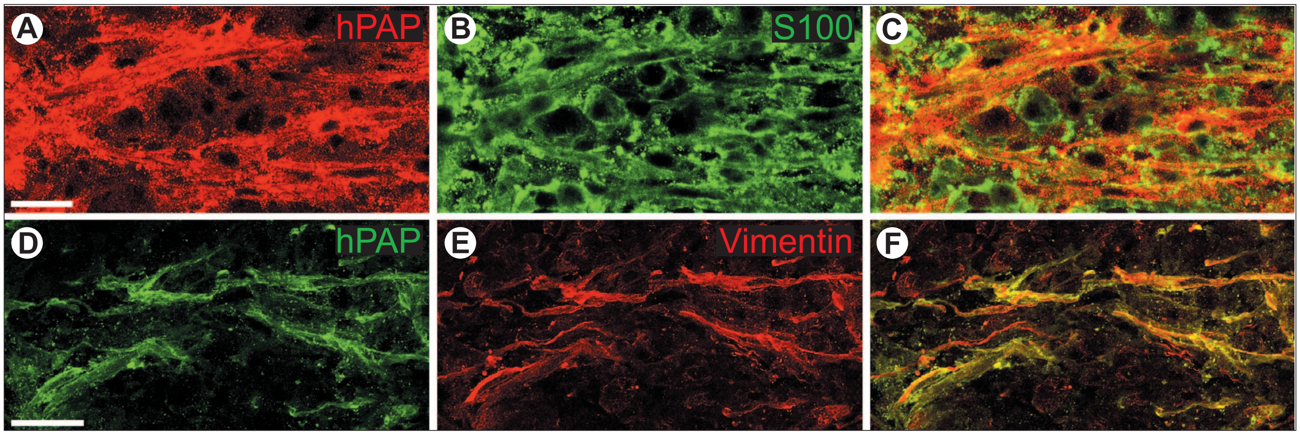

Additional figure 2

Expression of astrocytic markers by GDAs *in vivo*. At 4 days after injury and transplantation, the majority of $hPAP^+$ GDAs **(a,d)** within dorsal column lesion centers and margins display immunoreactivity for **(b)** S100 and **(e)** vimentin. **(c,f)** Sequential scanned confocal images through 2.5 μm depth show co-localization of **(c)** S100 and **(f)** vimentin with $hPAP^+$ GDAs. Scale bars represent 25 μm.
