# Supplementary material for: Astrocytes derived from glial-restricted precursors promote spinal cord repair
Source: J Biol. 2006 Apr 27;5(3):7. doi: 10.1186/jbiol35 (PMC1561531; doi:10.1186/jbiol35)
Supplement: Additional data file 3 — A figure showing misaligned host astrocytic processes in control lesions [file jbiol35-s3.pdf]

Additional data file 3

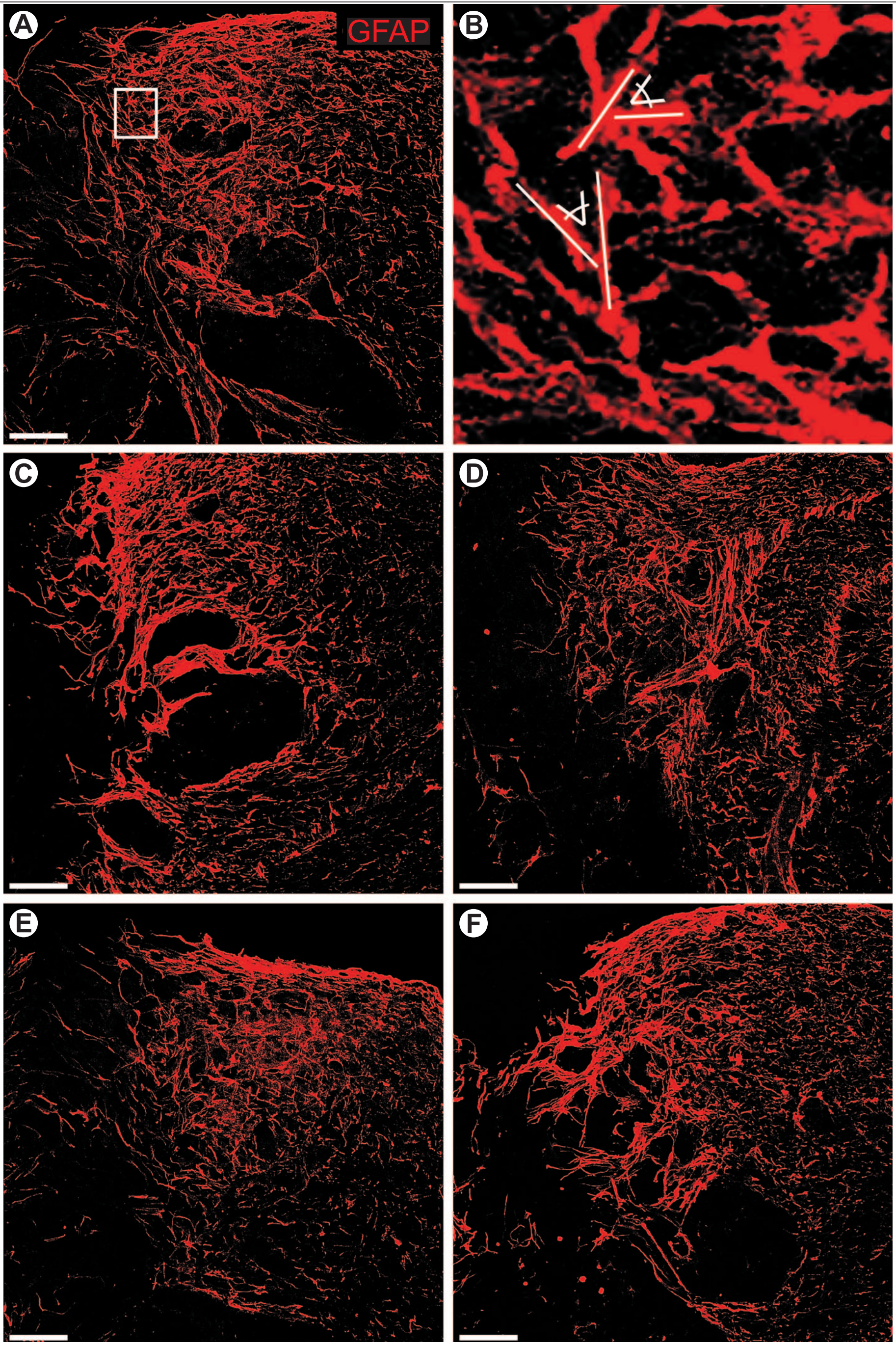

Additional figure 3

Misaligned host astrocytic processes in control lesions. **(a,c-f)** Confocal images of caudal lesion margins at 8 days after injury from three control animals showing dense mesh-works of hypertrophic GFAP$^+$ processes of host astrocytes, typical of forming glial scar tissue. **(b)** High-power image of boxed area in (a). GFAP$^+$ processes were randomly selected within the lesion margin and 'best fit' lines traced over them using Image Pro Plus software. Adjacent GFAP$^+$ processes were then identically traced and the angle between the paired lines calculated using Image Pro software. Quantitative analysis of host GFAP$^+$ astrocytic processes revealed an average angle of 59.4° (s.d. ± 22, median = 61°) between adjacent paired processes. Scale bars in (a,c-f) represent 100 μm.
