# Supplementary material for: Astrocytes derived from glial-restricted precursors promote spinal cord repair
Source: J Biol. 2006 Apr 27;5(3):7. doi: 10.1186/jbiol35 (PMC1561531; doi:10.1186/jbiol35)
Supplement: Additional data file 4 — A figure showing suppression of atrophy of axotomized red nucleus neurons by intra-spinal transplanted GDAs [file jbiol35-s4.pdf]

Additional data file 4

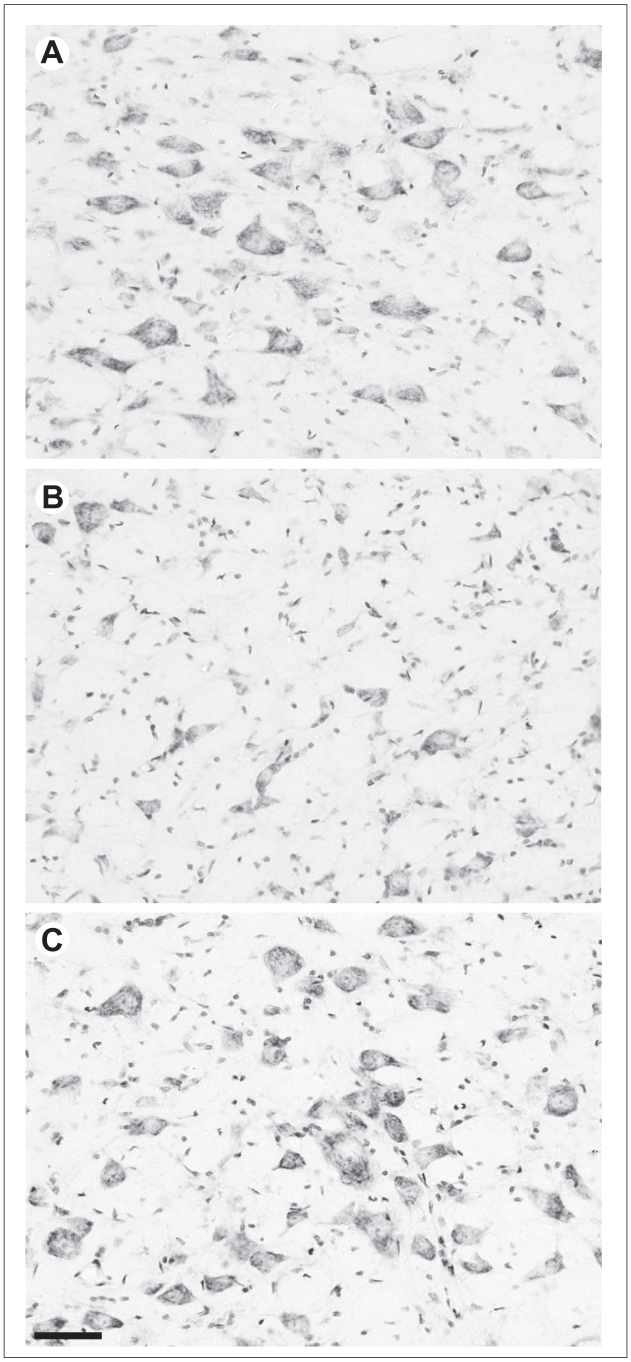

Additional figure 4
GDAs suppress atrophy of axotomized red-nucleus neurons. **(a,b)** Bright-field image showing cresyl violet stained neurons in the (a) uninjured right side and (b) injured left side red nuclei in a control, lesion + cyclosporine rat at 5 weeks after injury. Note in (b) that 48% of cresyl violet-stained neurons in the injured left hemisphere red nucleus undergo atrophy of their cell bodies. Images in (a,b) were obtained from right and left nuclei within the same 25 μm coronal tissue section. **(c)** GDA transplantation to the spinal cord rescues 65% of neurons that would normally undergo atrophy in the injured left side red nucleus. The image in (c) was obtained from the same rostro-caudal region of the red nucleus as the images shown in (a,b). The scale bar represents 100 μm.
